# Control Release Anesthetics to Enable an Integrated Anesthetic-mesenchymal Stromal Cell Therapeutic

**DOI:** 10.21767/2471-982X.100012

**Published:** 2016-08-02

**Authors:** T Maguire, M Davis, I Marrero-Berrios, C Zhu, C Gaughan, J Weinberg, D Manchikalapati, J SchianodiCola, H Kamath, R Schloss, J Yarmush

**Affiliations:** 1 Rutgers Department of Biomedical Engineering, Rutgers, The State University of New Jersey, Piscataway, New Jersey, USA; 2 BeauRidge Pharmaceuticals, LLC, New York, USA; 3 Department of Anesthesiology, New York Methodist Hospital, Brooklyn, New York, USA

**Keywords:** Controlled release, Local anesthetics, Liposomes, Alginate encapsulation, Molecular dynamic simulations, COMSOL model

## Abstract

While general anesthetics control pain via consciousness regulation, local anesthetics (LAs) act by decreasing sensation in the localized area of administration by blocking nerve transmission to pain centers. Perioperative intra-articular administration of LAs is a commonly employed practice in orthopedic procedures to minimize patient surgical and post-surgical pain and discomfort. LAs are also co-administered with cellular mesenchymal stromal cell (MSC) therapies for a variety of tissue regenerative and inflammatory applications including osteoarthritis (OA) treatment; however, LAs can affect MSC viability and function. Therefore, finding an improved method to co-administer LAs with cells has become critically important. We have developed a sustained release LA delivery model that could enable the co-administration of LAs and MSCs. Encapsulation of liposomes within an alginate matrix leads to sustained release of bupivacaine as compared to bupivacaine-containing liposomes alone. Furthermore, drug release is maintained for a minimum of 4 days and the alginate-liposome capsules mitigated the adverse effects of bupivacaine on MSC viability.

## Introduction

Perioperative intra-articular administration of LAs is a commonly employed procedure in orthopedic practices to minimize patient pain and discomfort [1]. LAs act directly on voltage-gated sodium channels and reversibly block the conductance in neurons [2]. However, despite their efficacy in pain control, LAs may also have detrimental off target effects on the surrounding tissues after intra-articular injection. Tissue reparative and protective mesenchymal stromal cells (MSCs) possess multi-lineage differentiation potential [3,4], immunomodulatory functions [4–6], are generally non-immunogenic [4,7,8], and are relatively easy to grow and expand in culture [9]. For these reasons, MSCs have become an attractive option for tissue engineering and regenerative medicine applications [9]. Cell therapies based on the surgical implantation or local injection of MSCs on an injured tissue site are a currently emerging tissue-repair approach for osteoarthritis and tissue injuries [10–12].

Therefore, recent studies including our own have looked more closely at the effect of LAs on MSCs. These studies demonstrate that LAs affect the proliferation capacity [2], differentiation potential, adherence phenotype [11,13], secretome [7,8], immunomodulatory function, and decrease the viability of MSCs [2,7,8,13,14] in a concentration, potency and time-dependent manner. Therefore, it is of pressing importance to develop cell therapy administration strategies that avoid compromising the integrity and potency of the therapy, but still deliver the necessary level of comfort to the patient.

A sustained release LA delivery model could enable the co-administration of LAs and MSCs without decreasing their anti-inflammatory or regenerative properties. Liposomes allow for this sustained release profile [15,16]. Liposome-LA constructs consist of a bilayer of lipids and contain LA within the interior space of the capsule. The structure of the liposome allows for the drug to be released, but at a slower rate than the harmful bolus dose, through leakage of the drug through the bilayer [15,16]. While the liposomes yield a decrease in release rate of LA to the cells, the drug is still released too quickly for clinical use of anesthetics. To combat this issue, we have developed a construct where bupivacaine containing liposomes are encapsulated in an alginate hydrogel [17,18]. Bupivacaine travels faster through the liposomes and slower through the alginate [17,18]. This tunable hydrogel encapsulated structure allows for control of the degradation and drug release profiles and can be designed to release sufficient and sustainable LA levels to minimize pain without harming therapeutic cell function.

## Materials and Methods

### Molecular dynamic simulations

Molecular Dynamics (MD) Simulations were performed using AMBER 14 installed on MAC OSX utilizing 4 cores. The default Lipid 14 force field was employed as part of AmberTools 14. The liposomal component was modeled as a lipid bilayer constructed using the CHARMM-GUI website as the GAFFlipid Molecule 1,2-dioleoyl-sn-glycero-3-phosphocholine (DOPC) where 37 water molecules per lipid was used in the CHARMM build with 64 lipids in the upper leaflet and 64 in the lower leaflet. The DOPC system was solvated with a 15 Å layer of TIP3 water both above and below the DOPC bilayer and neutralized via addition of 0.15M KCl via Monte Carlo ion placing method. The simulation system was rectangular with periodic boundary conditions employed. The CHARMM-GUI formatted PDB file was converted to the Lipid14 formatted PDB file, use the script charmmlipid2amber.py in AmberTools14 and the periodic box estimated in the x, y and z axes as 80, 80 and 70 Å respectively.

To equilibrate the Lipid/Water system, minimization for 5000 cycles at constant volume (no SHAKE), followed by two separate heating steps to 100 K then to 303 K (SHAKE employed), followed by a 500 ps production run to ensure the stability of the periodic boundary conditions. Finally, production molecular dynamics was run on the equilibrated system for 125 ns with temperature control using the Langevin thermostat and pressure controlled using the anisotropic Berendsen barostat. Stability of the system was determined by plotting the area per lipid using the cpptraj module of Amber 14 to make sure the area per ns had reached approximate zero order as well as inspection of electron density profile.

Bupivacaine structure was generated using ChemDoodle Software and saved in the protein data base format. The bond lengths of the bupivacaine were quantum mechanically optimized using the RED server to render them suitable for use in Amber MD GAFF force field 99SB. Approximately 50 bupivacaine molecules were placed at random in the pre-equilibrated structure of the DOPC/water system using the Molegro Molecular Viewer. The same equilibration steps were carried out as outlined above and the area per lipid and electron density profiles compared to the DOPC/Water system without bupivacaine.

### Chemicals and reagents

Bupivacaine and other chemicals were purchased from Sigma Aldrich (Oakville, Ontario, Canada), unless otherwise stated. All cell culture reagents were purchased from Life Technologies (Carlsbad, CA), unless otherwise stated.

### Liposomal formulation containing bupivacaine

The liposomes were formed using a dehydration-rehydration protocol. Briefly, hydrogenated soy phosphatidylcholine and cholesterol dissolved in chloroform were combined in a 25 mL round bottom flask at a 7:3 molar ratio and dried on a rotavap under vacuum. The resulting lipid film was re-suspended in distilled water and incubated for 2 hours at 55°C. The solution was then snap-frozen and lyophilized overnight.

Next, bupivacaine was loaded by re-suspending the lipid in 70 mg/mL bupivacaine-HCl. Liposomes were extruded through polycarbonate membranes (200 μm pore size) 21 times to create small unilamellar vesicles. The liposomes were then eluted through a Sephadex G-50 size exclusion column equilibrated with 0.9% saline.

### Alginate encapsulation of liposomes

Alginate solution was prepared by dissolving 2.2 g of alginic acid sodium salt (MW: 100,000-200,000 g/mol, G Content: 65%-70%, Sigma Aldrich) in 100 mL of ultra-pure water, using a heated magnetic stir plate at a temperature of 65°C. The solution was then filtered using a 45 mm syringe filter (0.22 μm pore size, FisherBrand). To create the anesthetic-alginate mixture, a 2-6 mL aliquot of the liposome dosage form (dependent upon the target pharmaceutical application and thus the dosage) of the anesthetic (5% w/v) is added to an alginate solution to yield a final volume of 10 mL with drug concentration range of 1.0%-3.0% (w/v) and a final alginate concentration of 2.2% (w/v).

This alginate solution was transferred to a 10 mL syringe (BD Biosciences), which in turn was connected to a syringe pump (KD Scientific, Holliston, MA). Alginate beads were generated using an electrostatic bead generator (Nisco, Zurich, Switzerland) at a flow rate of 40 mL/h, and an applied voltage of 6.5 kV, resulting in beads with a diameter of 200 μm. The beads were extruded into a 200 mL bath of CaCl_2_ (100 mM) (Sigma-Aldrich), containing 145 mM NaCl (Sigma-Aldrich), and 10 mM MOPS (Sigma-Aldrich) and are left to polymerize for 10 min at room temperature. Beads were transferred to a separate glass container, following the polymerization step. The beads were washed in DI water and resuspended in in water.

Distribution of the liposomes within the hydrogel was determined using the encapsulation of a fluorophore within the liposome, Z-section images of the 200 μm diameter beads were taken at 50 μm intervals (Zeiss 710 Confocal Microscope), to avoid multiple quantification of the same liposomal layer. Images were quantified using our in-house image thresholding, normalization and segmentation algorithms (Matlab). After the images were converted to black and white, and image normalization was performed, a color threshold for white pixels was applied to provide regions for subsequent image segmentation. The center of each segmented region was then determined in 3-dimensional space.

### Release of bupivacaine from the liposomes

Bupivacaine release from the liposomal-alginate hydrogel into PBS was measured experimentally. PBS samples were collected over time and bupivacaine concentration was measured using high-performance liquid chromatography. Samples were injected onto an 8 × 100 mm column (Radial-Pak 8NVCN, 4 M; Waters, Milford, MA). A mobile phase of 25 mM acetonitrile:phosphate buffer (75:25) with a pH of 4.0 was used, and absorption measured at a wavelength of 210 nm.

### Computational methods

COMSOL Multiphysics 5a (COMSOL Inc. Burlington, MA, USA) was used to model the fluid dynamics of bupivacaine release out of the liposomal-alginate hydrogel formulation. The model was conducted in a system composed of a 24 well-plate with transwell inserts, at fixed temperature with a no-flux condition on all interfaces except the insert-bottom well interface. Media was assumed to be an incompressible Newtonian fluid with a density of 1000 kg/m_3_.

An incompressible Navier-Stokes model was used, as well as a diffusion-convection model. Mesh optimization was performed for the simulations. A precondition iterative solver using a time stepping method was used, with a time step of 10s. Diffusivity of bupivacaine in media was 1E-10 mol/m^3^ [19]. Diffusivity of bupivacaine in the alginate-liposome system was determined by fitting, *via* objective minimization, the model’s concentration profile to experimental *in vitro* data. In the model system, the alginate-liposome drug mixture was treated as one geometry and placed in the transwell insert and the cells at the bottom of the well. In parallel, the bolus bupivacaine diffusion profile, in the 24 well-plate geometry, was tested by placing bupivacaine in media solution at several concentrations in the transwell insert and allowed to diffuse to the bottom well. The initial concentration of bupivacaine inside the alginate-liposome system or bolus dose ranged from 0.01-1 mM and the released concentration values were quantified at 0 hr, 1 hr, 2 hr, 4 hr, 12 hr, 24 hr, 48 hr, and 96 hr. To determine the concentration of bupivacaine in the bottom well, the concentration of bupivacaine at the cell surface and the concentration of bupivacaine released from the liposomal-alginate hydrogel system were determined by using COMSOL’s built-in surface integration and volume integration, respectively. Then, the concentration in the well was divided by initial concentration of drug in the transwell insert to obtain percent release of the system.

### MSC culture and treatment conditions

Human bone marrow-derived MSCs (Institute for Regenerative Medicine, Texas A&M College of Medicine) were cultured as previously described by Gray, with some modifications [7,8]. Briefly, MSCs were thawed at passage 2 and plated as a monolayer culture at 300,000 cells in T-175 flask (~1714/cm2) in a humidified 37°C, 5% CO_2_ incubator. Cells were cultured in Minimum Essential medium α containing no deoxy-or ribonucleosides, and supplemented with 10% fetal bovine serum (Atlanta Biologies), 2 mM L-glutamine, 1 ng/mL basic fibroblast growth factor, 100 U/mL penicillin and 100 mg/mL streptomycin. The cells were grown to 70% confluence, trypsinized, seeded into 24-well plates at 12000 cells/well (6000 cells/cm2), and allowed to attach overnight.

Transwell inserts (Corning) containing fresh media and 1 mM of bolus bupivacaine, bupivacaine- loaded liposomes, or bupivacaine- loaded liposomes embedded in alginate were placed in culture with MSCs and incubated over a period of 96 hours in a humidified 37°C, 5% CO_2_ incubator.

### Assessment of cell viability/proliferation

After 96 hours of culture in the presence of different bupivacaine delivery systems, the transwell inserts were removed, and the cell culture supernatants were replaced with medium containing CellTiter-Blue Cell Viability Assay reagent (Promega) according to the manufacturer’s instructions. The cells were then returned to the incubator for continued culture. The fluorescence of the wells was read at 4 hours using a microplate reader (DTX880 Multimode Detector, Beckman Coulter), returning the cells to the incubator between readings.

MSCs were fixed and stained for counting as previously described by Gray [7,8]. Briefly, supernatants were removed and replaced with 4% (w/v) paraformaldehyde (PF) to fix the cells. After 15 minutes of fixing at room temperature, enough 1× phosphate buffered saline (PBS) (Life Technologies) was added to each well to obtain 1% (w/v) PF and the cells stored at 4°C for further analysis. The fixed cells were transitioned to room temperature, washed 3 times with PBS for 5 minutes, incubated with 300 μL Hoechst stain (Molecular Probes) diluted 1:5000 in PBS for imaging. Stained nuclei were imaged at 4× using an inverted fluorescent microscope (IX81, Olympus) and counted using Slide Book 5.0 software (Intelligent Imaging Innovations) image analysis software. Cell count numbers were then used to normalize fluorescence intensity measurements obtained from the Cell Titer Blue assay.

### Statistics

Data points represent the mean ± the standard error of the mean (SEM) for the indicated number of independent observations (n). Statistical significance was determined using the Student’s t-test for unpaired data in Matlab. Viability/proliferation data are reported as fluorescence intensities divided by cell number. Statistical differences among the data were determined using analysis of variance (ANOVA) followed by Fisher’s least significant difference (LSD) post hoc analysis with a significance level of α=0.05 in Kaleida-Graph software version 4.1 (Synergy Software, Reading, PA, USA).

## Results

Currently available LAs have relatively brief durations of action. An ultralong-acting local anesthetic would benefit patients with acute and chronic pain with the added advantage of minimal toxicity to the local cellular environment and to co-administered cell therapies. In this formulation we utilize a microencapsulation approach to create small microbeads (~200 μm diameter) that are an alginate and anesthetic liposome composites. In order to provide a sustained release bupivacaine formulation which will provide a significantly prolonged release, we designed a new system which comprises an injectable/implantable unilamellar liposome containing bupivacaine, encapsulated within an alginate hydrogel gelling system. The novel combination of these two well-known technologies was designed to provide release of local anesthetic at a discrete site in a concentration effective to attenuate or relieve pain without affecting the function of co-administered or by-stander cells.

### Molecular dynamic simulations

**Figure 1:**
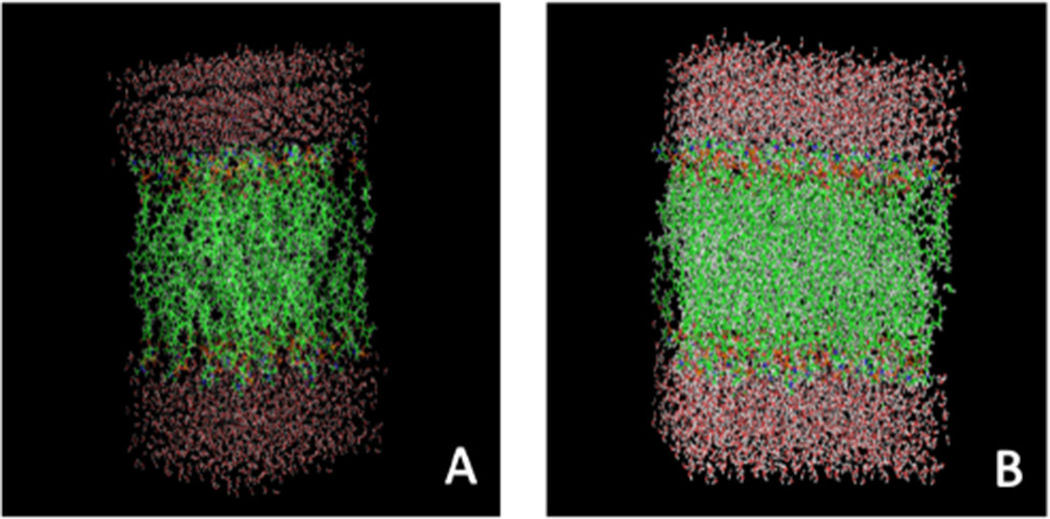
Molecular Dynamic simulation of DOPC water-bupivacaine system. A) Liposome layer folds correctly with hydrophobic and hydrophilic components. B) Water packed liposome model. DOPC lipid bilayer system is stable even after the addition of bupivacaine indicating intercalation among the lipid tails is a likely path for bupivacaine release. This may indicate that using different lipid/ head group combinations could alter bupivacaine release rate.

Prior to *in vitro* assessment of the formulation, soy phosphatidylcholine was first optimized using coarse grain modeling techniques (Figure 1). 1,2-Dipalmitoyl-sn-Glycero-3-Phosphocholine (DPPC) was used during the development phase of our model because there is a large amount of available experimental data regarding the structural properties of this phospholipid, allowing us to validate our model. Figure 1 illustrates coarse graining in our model as applied to DPPC. The choline moiety (N(CH_3_)_3_), the phosphate group (PO_4_), the glycerol ester linkage (COO), alkyl group (CH_X_) are each represented as single interaction sites. Our results indicate that the lipoprotein will form stable vesicular structures around the bupivacaine. Furthermore, we can achieve 95-97% loading.

### Liposomal-hydrogel characterization

**Figure 2:**
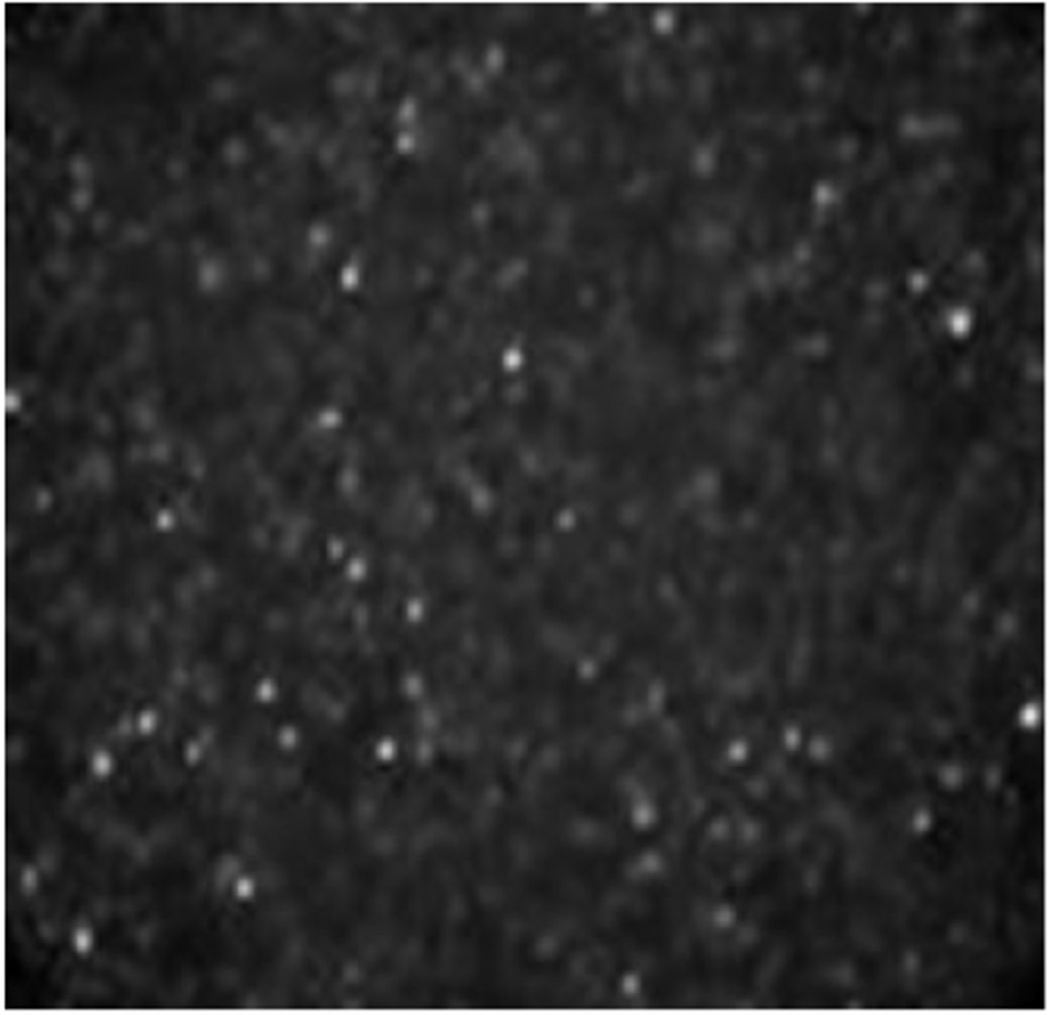
Fluorescent image of liposomes in alginate. The image is a representation of a z-section. As can be seen, a relatively homogenous distribution of liposomes is contained within each microbead. Microbeads were 200 μm +/− 5% in diameter.

**Figure 3:**
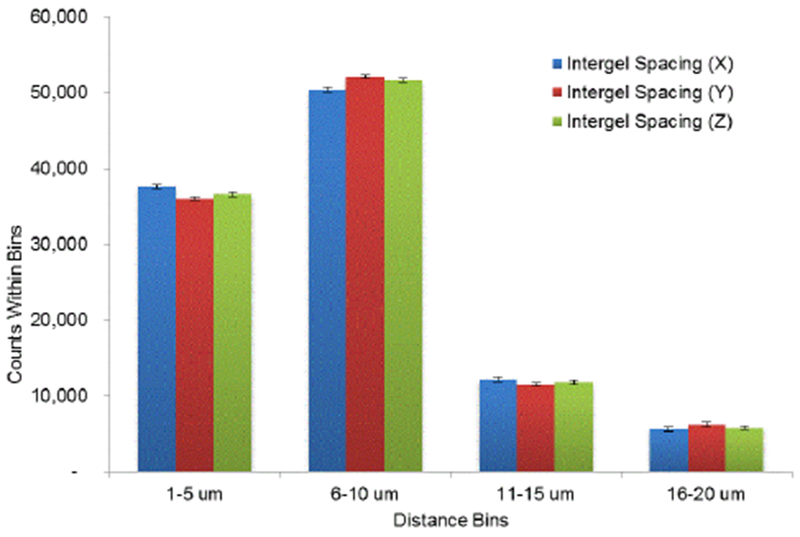
Assessment of liposomal distribution within alginate microbeads. Liposomal distribution within the capsules was determined via statistical analysis of between-centroid within each microbead.

Following verification of liposomal-bupivacaine interaction and formation, we then prepared and characterized the formulation contained within a fast set alginate gel [20–24] (Figure 2). The distance between the centroid-of-mass of the fluorescent particles was then measured via an in-house Matlab algorithm (Figure 3) and demonstrated a relatively even distribution.

### Release of bupivacaine from the liposomes

**Figure 4:**
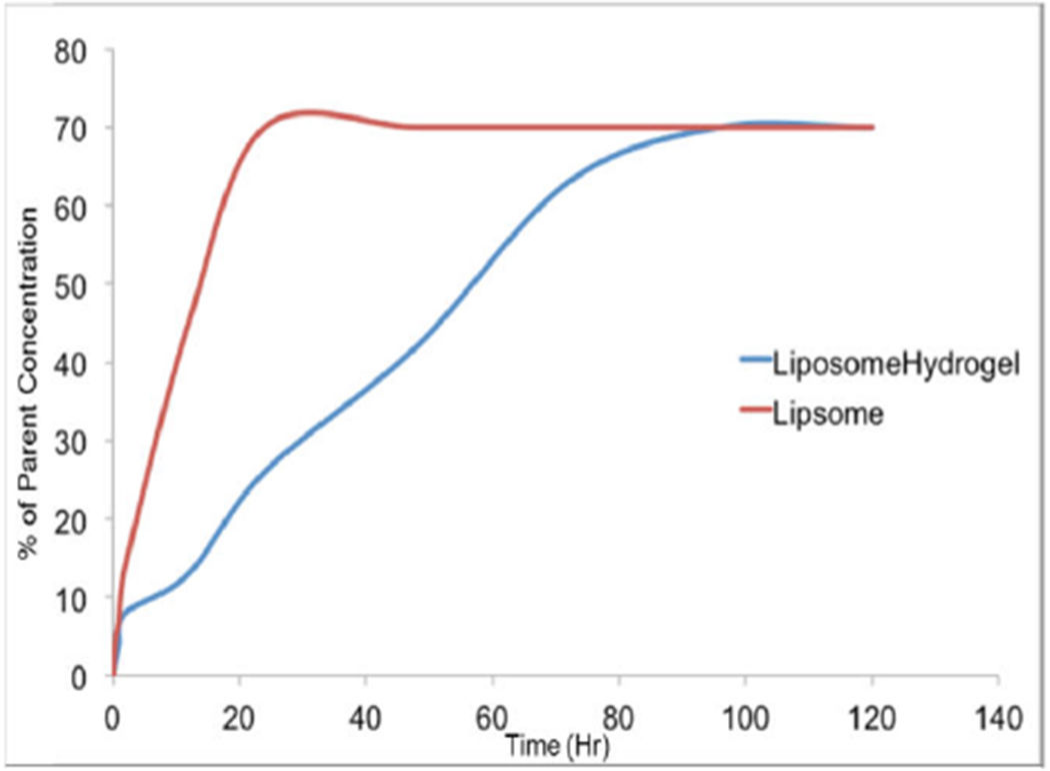
Control release of bupivacaine from liposomal-hydrogel constructs. Release of bupivacaine into an *in vivo* relevant medium (cell-culture media) was determined using an LCMS protocol, optimized for bupivacaine detection.

The retention time of bupivacaine in liposomes was found to be approximately 4.7 min in the column. As indicated in Figure 4, encapsulation of liposomes led to a sustained release of bupivacaine as compared to bupivacaine-containing liposomes alone. In addition, while liposome vesicles released the drug by 24 hours, release in the liposomal-alginate hydrogel construct was maintained for a minimum of 4 days.

### Control release simulation

**Figure 5:**
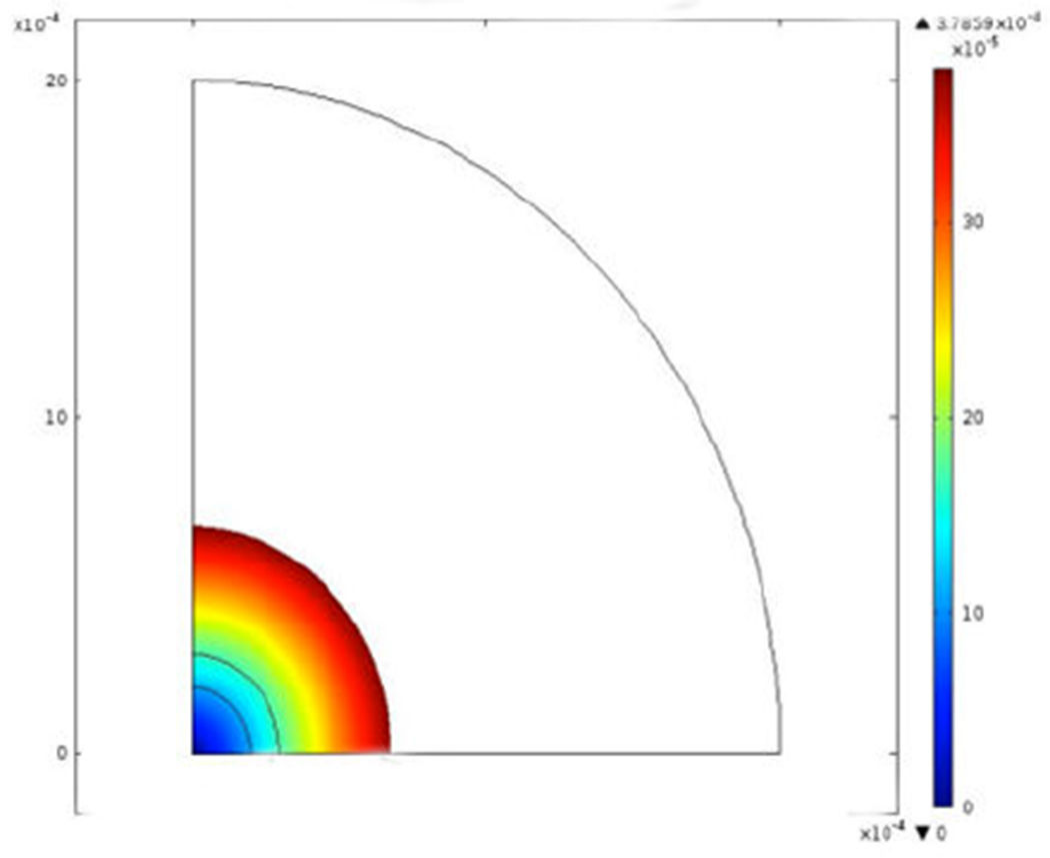
CFD assessment of drug release from liposomal-hydrogel formulation. Figure demonstrates a CFD assessment of drug release from a liposomal formulation alone at 24 hours.

To close the loop on the hypothesis that one is able to control local concentrations of analgesic to potentially mitigate adverse cellular responses in the presence of bupivacaine, we used COMSOL to understand the sustained release profiles of both the liposomal and liposomal-hydrogel formulations within the liposome, and at a distance outside of the liposome (Figure 5).

As indicated in Figure 5, not only is there a prolonged release of bupivacaine, one is also able to modulate the maximal concentration of analgesic at a given distance by tuning the dominant parameters of the concatenated formulation. As can be seen in Figure 5, bupivacaine diffuses within the liposome space and then is completely released from the liposome within 24 hours.

Prior studies including our own [7,8,19], demonstrated that after 48 hours of continuous exposure, a bolus dose of 0.1 mM bupivacaine yielded a 90% cell viability (Table 1). 90% cell viability was considered desirable to remain consistent with pharmaceutical industry viability standards [25–27]. The liposomal-alginate hydrogel formulation should therefore have a release profile that does not exceed 0.1 mM at a given time to ensure high cell viability.

**Figure 6:**
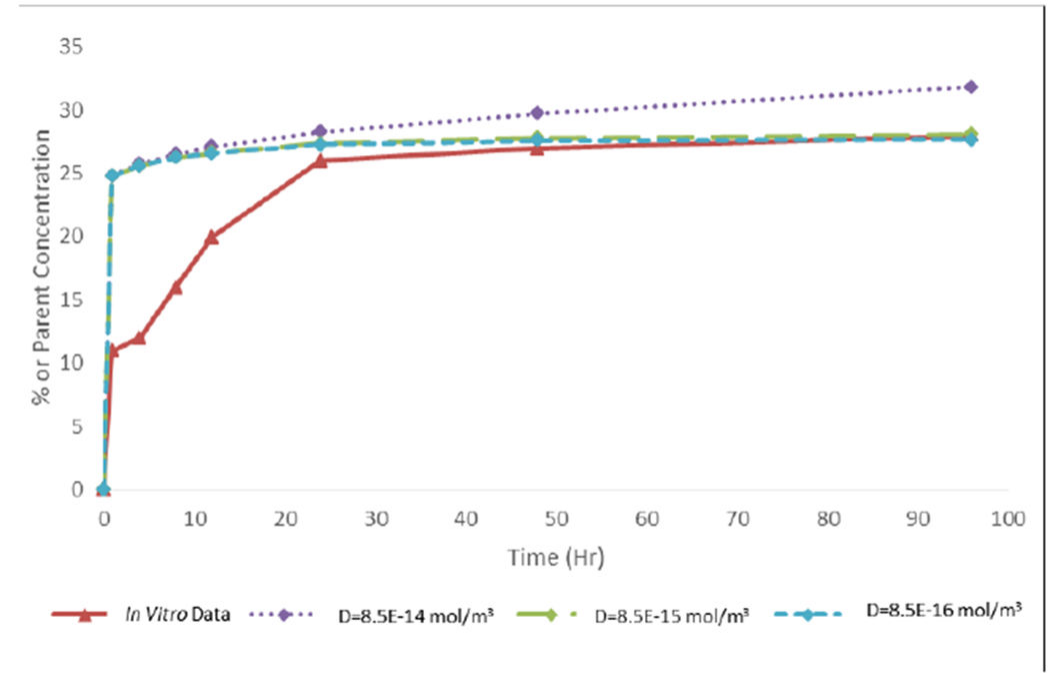
Diffusivity of bupivacaine from liposomal-hydrogel formulation. Comparing *in vitro* bupivacaine release data to model output at various diffusivity values. A final diffusivity value was determined to be 8.5E-15 mol/m^3^ for 1 mM bupivacaine in 24 well-plate transwell geometry.

Using the *in vitro* release data (Figure 4), the diffusivity of bupivacaine from the liposomal-alginate hydrogel system was determined. COMSOL Multiphysics was used to determine that the overall diffusivity is 8.5E-15 mol/m^3^ (Figure 6) by minimizing sum of squares between the experimental *in vitro* data and the theoretical modeled data.

**Figure 7:**
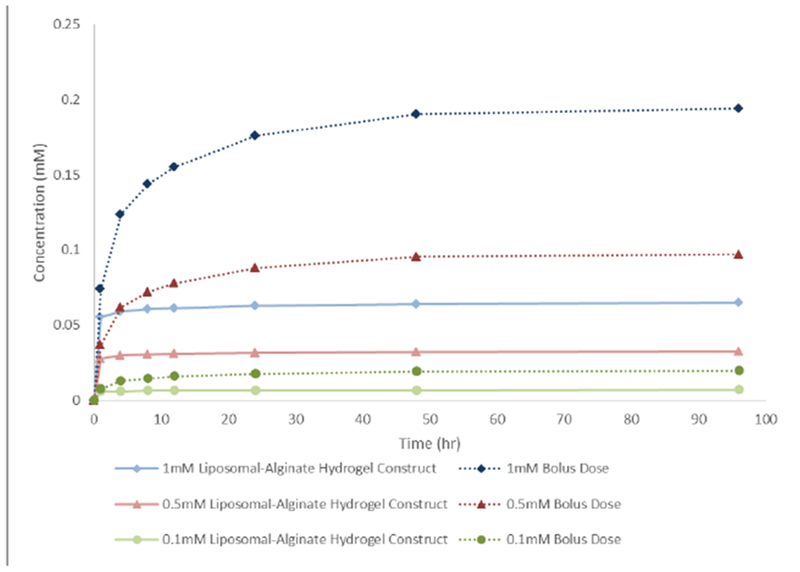
Simulated *in vitro* bupivacaine release profile over time. Model output comparing the transwell alginate-liposome formulation with the transwell media-bolus concentration at different initial bupivacaine concentrations. The alginate-liposome system decreased the release profile of bupivacaine.

Using this diffusivity, a model was established for the entire system. To test to difference between the transwell liposomal-alginate hydrogel system and a transwell bolus bupivacaine dose, COMSOL Multiphysics was used to model both cases, with and without the alginate component. Figure 7 shows that the liposomal-alginate hydrogel construct decreased the release rate for the three concentrations of bupivacaine tested. According to our model system, an alginate-liposome composition with 1 mM bupivacaine will release less bupivacaine over time than a 0.1 mM bolus dose, which was established as the cut off concentration to maintain high cell viability (Table 1).

### Assessment of cell viability/proliferation

**Figure 8:**
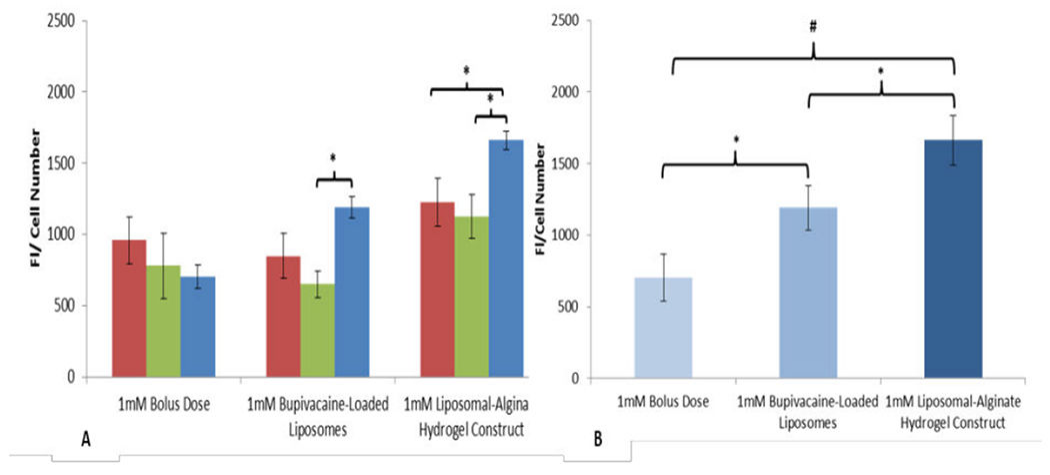
Liposomal-hydrogel formulation mitigates adverse cellular viability response. A) The bar heights represent the fluorescence intensities (FI) of reduced CellTiter-Blue reagent normalized by cell number after 24 (red bars), 48 (green bars), and 96 (blue bars) hours of incubation with MSCs treated with 1 mM bolus bupivacaine, liposomal bupivacaine, or liposomal-hydrogel formulation. B) After 96 hours in culture there is a significant protection of cell viability in the liposomal-alginate hydrogel construct conditions.

Lastly, to corroborate the predictions of the controlled release simulations, we investigated the effect of 1 mM bolus bupivacaine, bupivacaine-loaded liposomes, and the liposomal-hydrogel bupivacaine formulation on MSC viability using CellTiter-Blue Cell Viability Assay. As indicated in Figure 8, in all time points, MSCs showed an improved cell viability response in co-culture with the liposomal-alginate hydrogel formulation compared to bolus bupivacaine dose or bupivacaine-loaded liposomes.

The bar heights in the Figure 8 represent the fluorescence intensities (FI) of reduced CellTiter-Blue reagent normalized by cell number after 96 hours of incubation with MSCs treated with 1 mM bolus bupivacaine, liposomal bupivacaine, or liposomal-hydrogel formulation. The data are the mean ± SEM of n=6 independent observations (N=2 experiments). (*) represents the Statistically different (p ≤ 0.05) among treatment conditions. (^+^) represents the Statistically different (p ≤ 0.0001) between bolus bupivacaine and liposomal-hydrogel bupivacaine formulation.

## Discussion

LAs are drugs which provide local numbness and/or analgesia. While compounds utilized as general anesthetics reduce pain by producing a loss of consciousness, LAs act by producing a loss of sensation in the localized area of administration. LAs are a family of drugs with a long history of providing local anesthesia for surgery and painful procedures [28,29]. Given the emerging use of cellular therapeutics such as MSC for tissue regenerative applications, a number of studies have explored the effects of LA on MSC viability and regenerative function [2,7–9,11–14]. Among these, our studies demonstrated that bupivacaine reduces anti-inflammatory MSC function and this functional loss is sustained even after the drug is removed [30]. Therefore, finding an improved method to co-administer LAs with cells has become critically important. The results of the present studies indicate that an alginate-liposome-bupivacaine construct can effectively control LA release into the environment.

In general, LAs are recognized as providing a rapid onset, but a relatively short duration of action [31–34]. Several approaches for provision of multiday duration local analgesia have been attempted. Exparel (liposomal bupivacaine) was approved for provision of postoperative pain relief for a limited number of surgical procedures [17]. Exparel consistently provides 24 hour pain reduction but has failed to provide longer-term reduction (the target was 72 hours of pain reduction). In addition, Exparel must be handled as a strict cold-chain product in that freezing or higher-than-room temperature storage for even short periods of time causes dose-dumping which would release toxic concentrations of bupivacaine. Posidur (bupivacaine contained in a substituted sugar matrix) has been studied extensively and delivers bupivacaine over 72 hours. Posidur may only be used for small surgical procedures since the vehicle used for storage and delivery (an alcohol) causes local tissue toxicity, limiting injection volumes to approximately 5 mL.

The unique physical properties of hydrogels have sparked particular interest in their use in drug delivery applications. Their highly porous structure can easily be tuned by controlling the density of cross-links in the gel matrix and the affinity of the hydrogels for the aqueous environment in which they are swollen. Their porosity also permits loading of drugs into the gel matrix and subsequent drug release at a rate dependent on the diffusion coefficient of the small molecule or macromolecule through the gel network. Indeed, the benefits of hydrogels for drug delivery may be largely pharmacokinetic especially since a depot formulation is created from which drugs slowly elute, maintaining a high local concentration of drug in the surrounding tissues over an extended period, although they can also be used for systemic delivery. Hydrogels are also generally highly biocompatible, as reflected in their successful use in the peritoneum and other sites *in vivo.* Biocompatibility is promoted by the high water content of hydrogels and the physiochemical similarity of hydrogels to the native extracellular matrix, both compositionally (particularly in the case of carbohydrate-based hydrogels) and mechanically.

In order to optimize our formulation we used molecular dynamic simulation. Molecular dynamics simulation has been used extensively to investigate pure lipid bilayers in the last decade and, due to the excellent experimental agreement, is now being used by many groups to probe more complex systems with potential biomedical applications. The studies can be divided roughly into two categories: high-resolution and low-resolution models. High-resolution or atomistic models are based on a realistic representation of membrane geometry and energetics and typically account for the motion of every membrane and solvent atom. This approach has been used to simulate the aggregation of aqueous solutions of lipids into bilayers and vesicles and to observe the early stages of spontaneous phase separation in multi-lipid bilayers. The detail that makes high-resolution models so realistic also makes them extremely intensive computationally, precluding their application to problems involving large conformational changes or long time scales. Low-resolution models are based on a coarse-grained representation of lipid geometry and energetics, typically accounting for the motion of groups of atoms, as opposed to individual atoms. Sometimes the motion of the solvent atoms is ignored to enhance computational efficiency; instead the effects of solvent atoms are included implicitly through the use of effective potentials, or potentials of mean force. Application of the coarse-graining approach to the modelling of membranes has grown substantially. Here we applied a newly developed coarse-grained model. The design and optimization of the proposed dual-fusion liposomes was facilitated by an experimentally informed computational approach. Discontinuous molecular dynamics (DMD) simulation was applied to “LIME”. The simulations are used to help determine the best combination(s) of structural design parameters for this therapeutic modality, including: the mole fractions, compositions and tail lengths of the two lipids. Overall, the simulations are utilized as a predictive tool to help accelerate and optimize the design of the liposome.

In addition, COMSOL modeling was used to understand the diffusivity of bupivacaine from the liposomal-alginate hydrogel construct. This model determined that our formulation could enable long term release of lower concentrations of bupivacaine to MSCs, or other proximate cell types, which would therefore preserve the viability of the cells. Previous studies performed by our group suggest that MSCs are 90% viable after 48 hours of culture in the presence of 0.1 mM bolus bupivacaine; therefore, for this system, the goal was to ensure the cells maintained a high viability (90% viable cells compared to basal medium exposure) by controlling the concentration the cell “sees”, or the cell apparent concentration. The diffusion model for the liposomal-alginate hydrogel system showed that at the highest starting dose of 1 mM bupivacaine in the liposomes, a cell apparent concentration of less than 0.1 mM at a given time point was seen, enabling a cell viability of at least 90%.

The diffusivity of bupivacaine from the liposomal-alginate hydrogel construct was determined using *in vitro* LC-MS release data (Figure 6). While individual diffusivities of bupivacaine from alginate and from liposomes have been previously determined, the combination of our system has never been evaluated, and therefore the value needed to be determined experimentally. The model system diffusion mimics the *in vitro* data after 24 hours. For the purpose of this long term release study, the longer time points are more clinically relevant than the initial time points, and therefore, those *in vitro* concentrations were used to determine the diffusivity value. The model system simulation and the *in vitro* data differ before 24 hours, with a large bupivacaine release in the model system, but a more gradual release in the *in vitro* data. This discrepancy may be due to the simplicity of the model system, which does not take into account binding and interactions between the drug and alginate and lipids in the system, which may be increased at the initial time points. In addition, this is a phase model and therefore, does not take into account the arrangement of liposomes in the alginate and instead treats the system as one unit.

For simplicity, this model system does not take into account cell uptake of bupivacaine, and therefore, the geometry of the modelled system is a key parameter in determining the overall diffusion of the system. For instance, this model system set up in a 96 well-plate transwell system led to bupivacaine equilibrium within the first few hours, whereas in a larger geometry system, equilibrium was never reached (unpublished observations). Therefore, the geometry of the *in vitro* data and the model system must be kept constant in order to find a drug or compound’s diffusivity value.

Understanding the diffusion pattern of bupivacaine out of the liposomal-alginate hydrogel construct allows for easy modifications to yield a faster or slower diffusion of drug out of the system and towards the cells. These modifications include liposome concentration within the alginate, drug concentration within the liposome as well as within the entire system, alginate stiffness, and distance from intended uptake. Moreover, the diffusivity allows for easy dosing and scale-up for clinical use. Knowing the geometry and the cell apparent dose needed for analgesic effect, the correct liposomal-alginate hydrogel formulation can be designed.

To corroborate the modelling data, *in vitro* studies were performed using monolayer MSCs treated with bolus dose, bupivacaine-loaded liposomes, or the liposomal-alginate hydrogel construct. The *in vitro* studies indicate that after four days in continuous culture, the constructs yielded significantly greater MSC viability than either the bupivacaine-loaded liposomes alone or the bolus dose (Figure 8). Encapsulating bupivacaine in the liposomal-alginate hydrogel construct helped mitigate the LA adverse effects previously seen in MSCs. Further studies to investigate the therapeutic efficacy of MSC in the presence of the liposomal-alginate hydrogel formulation are currently in progress.

The significance of our studies lies in the target goal of providing a multi-day prolonged period of pain mitigation in post-operative surgical patients and for procedures which co-administer LA with cell preparations such as MSC. Current standards of pain mitigation are limited to 6 hour windows of efficacy and thereby require multi-day dosing both in inpatient and outpatient settings. Multiple dosing administered through either liquid dosage from injections, applied locally, or through oral solid dosage forms such as oxycodone, have adverse side-effects such as the potential for tolerance, addiction, or limited efficacy leading to a delay in the ability to rehabilitate patients following surgery. By providing a controlled release product that can be administered immediately after surgery, prior to the completion of surgery, in the context of cellular therapeutics, and last for multiple days, can potentially mitigate the risks associated with pain treatment.

Taken as a whole, evidence from the clinical studies of liposomal bupivacaine suggests this local anesthetic formulation may be a useful component of multimodal analgesic regimens for managing postsurgical pain in select patients, with the potential to reduce opioid use and opioid-related adverse drug events (ADEs) in the postsurgical setting. As previously mentioned, [7,8,11,30] integration of an analgesic effect with a potential cell therapy requires additional formulation approaches to mitigate the effects on the cell therapy so as to not inhibit its therapeutic effect. Using a long release hydrogel-formulation has the potential to fit these criteria.

As with bupivacaine, appropriate use of hydrogel-liposomal formulations of LA to optimize clinical effects, economic implications, and patient tolerability will depend on appropriate patient selection, practitioner training and institutional protocols. As a component of a multimodal analgesic regimen, a hydrogel-liposomal formulation of bupivacaine represents a new approach to extending the duration of post-surgical analgesia. Further *in vitro* release studies along with MSC or other cell culture functional studies are needed to demonstrate the efficacy of our approach. In addition *in vivo* studies across a range of surgical settings should help clarify the most appropriate roles for this prolonged-release formulation of bupivacaine.

## Figures and Tables

**Table 1:** Effect of bupivacaine concentrations on MSC viability. Percentage of viable cells after exposure to medium containing 0.01 mM, 0.1 mM, 0.5 mM and 1 mM of bupivacaine normalized to basal medium control (0 mM) with standard error. Data represents 3-4 independent experiments.

Concentration	0 mM	0.01 mM	0.1 mM	0.5 mM	1 mM
% Viability at 24 hours	100 ± 4%	86 ± 3%	70 ± 3%	64 ± 0%	43 ± 2%
% Viability at 48 hours	100 ± 4%	105 ± 4%	90 ± 2%	62 ± 35	38 ± 4%
